# Stone Between Crossed Paths: Retrograde Intrarenal Surgery for Isthmic Calculus in the Setting of Renal and Gut Malformations

**DOI:** 10.7759/cureus.110961

**Published:** 2026-06-16

**Authors:** Prajeeth Reddy, Venkata Ramana Korrapati, Roshan Yedulla Reddy, Hariharasudhan Sekar, Sriram Krishnamoorthy

**Affiliations:** 1 Urology, Sri Ramachandra Institute of Higher Education and Research, Chennai, IND

**Keywords:** congenital renal anomalies, horseshoe kidney, intestinal malrotation, isthmic renal calculus, retrograde intrarenal surgery (rirs)

## Abstract

Horseshoe kidney is the most frequent type of renal fusion anomaly, and it often has associated changes in the orientation of the kidneys, anomalous vascular supply, and poor urinary drainage, as well as an increased risk of nephrolithiasis. Horseshoe kidney occurs in conjunction with intestinal malrotation very infrequently, and the anatomical and technical difficulties are significant because of the distorted anatomy of the kidney and the abnormal bowel position; therefore, careful preoperative planning and an individualized surgical strategy are needed for the management of renal calculi in these patients.

A 63-year-old man with a six-month history of left loin pain, but no fever, dysuria, hematuria, or evidence of urinary tract infection, was found to have a horseshoe kidney with a 2.4 × 1.2 cm calculus in the renal isthmus on cross-sectional contrast-enhanced CT, as well as intestinal malrotation with abnormal bowel orientation and mesenteric vascular relationships. Given the complicated anatomy and risk of percutaneous access, the patient underwent retrograde intrarenal surgery with flexible ureteroscopy and holmium laser lithotripsy, with successful complete stone clearance and no intraoperative or postoperative complications. This case shows the value of performing an extensive anatomical evaluation before creating a plan tailored to a specific patient when treating patients with multiple congenital malformations. Additionally, this case shows that retrograde intrarenal surgery is a safe and effective way to minimally invasively treat complicated renal calculi inside the kidney when the anatomic space is difficult to access.

## Introduction

Horseshoe kidney is the most common renal fusion anomaly, which occurs in about 0.25% of the general population with a male-to-female ratio of 2:1 [1]. It is a congenital malformation caused by abnormal embryological development during the fourth to sixth weeks of gestation, when the metanephric blastemas fuse across the midline, usually at the lower poles [2]. Horseshoe kidney is characterized by malrotation of the renal units, a high insertion of the ureters, and an abnormal pattern of vascular supply, which can lead to multiple urological complications.

Horseshoe kidney patients have a much higher incidence (15%-60%) of nephrolithiasis compared to the general population [3], which is likely caused by incomplete renal rotation, altered urine transport, and urinary stasis due to ureteropelvic junction obstruction. Stones in these patients are technically challenging to manage because of the abnormal ureteral course and altered collecting system anatomy.

The occurrence of intestinal malrotation in conjunction with a horseshoe kidney is an extremely rare combination, seen in less than 1% of cases [4,5], and this association is suggestive of complex embryological malformations affecting multiple organ systems during critical periods of pregnancy [6]. In a rare case of a large isthmus calculus in a patient with a horseshoe kidney and intestinal malrotation, we report the successful treatment with retrograde intrarenal surgery, emphasizing the need for individualized treatment plans for complex congenital anomalies.

## Case presentation

A 63-year-old male patient presented with a six-month history of persistent left lumbar pain without associated fever, dysuria, or hematuria. The patient had no prior history of known congenital abnormalities, nephrolithiasis, or urinary tract infections. Physical examination revealed mild left loin tenderness without palpable masses.

Laboratory investigations demonstrated normal serum creatinine (1.1 mg/dL), and urine culture showed no growth. Contrast-enhanced CT of the abdomen revealed that both renal lower poles were seen pointing medially and were fused across the midline by an isthmus of functioning renal tissue. The right ureter was noted arising medially from the right moiety, and the left ureter from the left moiety and the isthmus. A calculus measuring 2.4 x 1.2 cm with maximum attenuation of +1402 HU was located in the calyceal group of the isthmus (Figures 1A-1C). Imaging also incidentally revealed intestinal malrotation, with the D3 segment of the duodenum not crossing the midline, the duodenojejunal flexure was noted on the right side, and an abnormal superior mesenteric artery/superior mesenteric vein relationship. A mesenteric "whirl" around the superior mesenteric artery was seen. The ileocecal junction was noted in the midline at the level of the umbilical region (Figures 2A, 2B).

**Figure 1:**
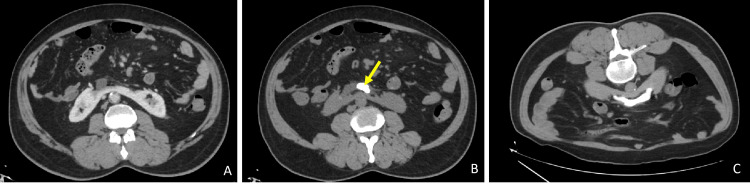
Imaging Representation of the Horseshoe Kidney with Isthmus Calculus (A) Contrast-enhanced CT of the abdomen (nephrogenic phase) showing a horseshoe kidney.  (B) Plain CT showing radio-dense calculus measuring 2.4 x 1.2 cm in the isthmus (yellow arrow). (C) Prone CT showing delayed phase, showing contrast excreting into the left ureter from the left moiety and isthmus.

**Figure 2:**
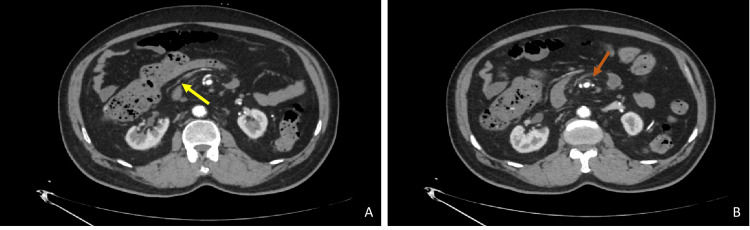
Imaging Representation of Intestinal Malrotation (A) Contrast-enhanced CT (CECT) of the abdomen (arterial phase) showing the ileoceacal junction in midline (yellow arrow). (B) CECT of the abdomen (arterial phase) showing the bowel swirling around the superior mesenteric artery (orange arrow).

Given the stone size, symptomatic presentation, and anatomical complexity, the patient was counselled regarding treatment options and elected to proceed with retrograde intrarenal surgery for stone management.

Ureteroscopy was performed using a semi-rigid 8/9.8-French ureteroscope to assess the ureter and place a safety guide wire. The left ureter was cannulated successfully, and a guidewire was placed into the left renal pelvis under fluoroscopic guidance, and retrograde pyelography was done. The ureter appeared normal in calibre; hence, a 10/12-French flexible and navigable suction ureteral access sheath was placed over the guide wire to facilitate the digital flexible ureteroscope. The stone was identified in the isthmus, which was connected to the left collecting system (Figures 3A-3C).

**Figure 3:**
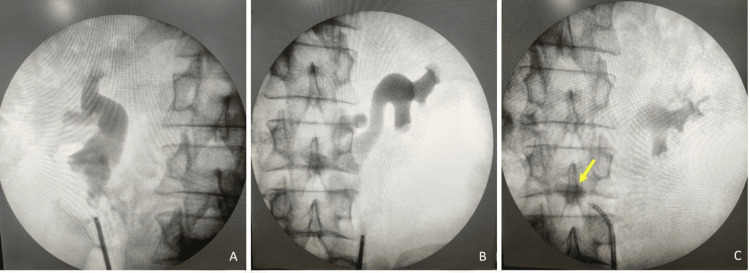
Intraoperative Retrograde Pyelography (A) Right collecting system in retrograde pyelogram. (B) Left collecting system in retrograde pyelogram. (C) Calculus with a flexible ureteroscope (yellow arrow).

The stone was completely dusted using a 272 Micron holmium laser fibre at 1.0 J and 10 Hz.

After confirming complete stone removal through fluoroscopic images and a complete review of the entire collecting system, a 6Fr/26 cm double J stent was placed to ensure adequate drainage (Figure 4).

**Figure 4:**
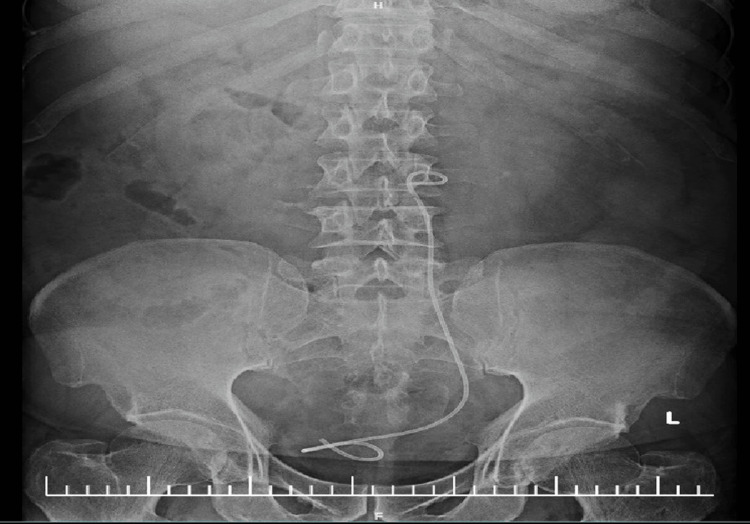
Postoperative Imaging Postoperative X-ray of the kidney and urinary bladder showing left double J stent in situ.

Considering the complex anatomy and stone burden, the total laser fragmentation time was approximately 30 minutes, and the procedure was completed without intraoperative complications. The fluid balance remained stable throughout the case, and essential blood loss was minimal.

The postoperative period was uncomplicated, and the patient was discharged on the first postoperative day with adequate pain medication, antibiotics, and instructions for stent care. He was educated about anticipated stent-related symptoms and directed to follow up.

At the two-week follow-up, the patient reported that his flank pain had completely resolved, and the ureteral stent was well tolerated with only mild frequency and urgency, which was expected as a temporary side effect due to the stent.

The ureteral stent was removed after three weeks postoperatively via flexible cystoscopy, which was well tolerated as an office procedure.

Following up at one month, the patient had complete resolution of all his presenting symptoms. His quality of life had improved significantly, and he expressed high satisfaction with the treatment outcome.

## Discussion

The management of renal stones (calculi) in horseshoe kidneys presents with some technical difficulties, especially when there are other abnormalities related to anatomy, like intestinal malrotation [7]. This case illustrates the complications of making surgical decisions with these two particular diagnoses; it also emphasizes the necessity of performing a thorough preoperative evaluation.

One in every 400 to 600 individuals with a horseshoe kidney has an increased risk of urolithiasis (kidney stones) due to poor drainage, recurrent infections, and anatomical distortion. Malrotation of the intestines adds to the difficulties in determining proper access points for percutaneous instrumentation due to the possible location of the bowel in relation to normal placement, which will also create risk to the procedure.

Percutaneous nephrolithotomy is the gold standard for large-volume renal calculi but has major limitations in horseshoe kidneys [8]. The altered renal anatomy, with anteriorly rotated collecting systems and closeness to critical structures including the aorta, the vena cava, and the improperly positioned colon, makes safe percutaneous access extremely difficult. The associated intestinal malrotation in our case increased these risks since the malpositioned colon might potentially be in the conventional percutaneous access path.

Retrograde intrarenal surgery emerged as the best option due to its ability to navigate the complex collecting system anatomy while avoiding percutaneous difficulties [9]. The flexible ureterorenoscopic procedure allows for direct visualization and accurate stone disintegration with laser lithotripsy, but complete stone clearance may necessitate staged procedures [10-13].

Preoperative planning with advanced imaging, including CT urography and possibly MR urography, is essential for accurately characterizing both renal and intestinal anatomy and for selecting the optimal surgical approach while minimizing surgical morbidity [14]. Based on our experience, we have found that developing an individualized plan for each patient's unique anatomical variations maximizes the potential for favorable outcomes in complicated urological cases [15].

To our knowledge, reports describing the management of an isthmic calculus in patients with concomitant horseshoe kidney and intestinal malrotation are extremely limited, and this case highlights the potential role of retrograde intrarenal surgery as a minimally invasive treatment option in this complex anatomical setting.

Take-home messages

Retrograde intrarenal surgery represents a safe, feasible, and effective minimally invasive therapeutic option for the management of complex renal calculi in anomalous kidneys, even in the setting of coexisting congenital abnormalities such as a horseshoe kidney and intestinal malrotation. 

Comprehensive preoperative anatomical delineation using advanced cross-sectional imaging is indispensable in patients with congenital renal anomalies, as aberrant renal orientation, vascular anatomy, ureteric course, and bowel positioning critically influence surgical planning, access, and procedural safety.

Individualized, anatomy-driven treatment strategies are paramount in complex urolithiasis associated with congenital malformations, and endoscopic approaches such as retrograde intrarenal surgery may offer significant advantages by reducing the potential morbidity and technical challenges associated with percutaneous interventions in distorted anatomical settings.

## Conclusions

This clinical case illustrates how retrograde intrarenal surgery is a good alternative for treating large kidney stones in the isthmus of a horseshoe kidney, particularly when intestinal malrotation is also present, because the retrograde approach avoids the technically challenging and potentially dangerous percutaneous nephrolithotomy, which is much more difficult in a horseshoe kidney because of the anatomical variations. Retrograde techniques through the natural urinary tract pathway are beneficial because they minimize complications related to misplaced bowels, reduce surgical trauma and the risk of damage to the bowel, and result in faster recovery from surgery. Comprehensive preoperative imaging is essential to delineate these anatomic variations and guide selection of the optimal surgical approach for each patient. Furthermore, with the continued development of endoscopic technology and techniques, retrograde intrarenal surgery will become increasingly favored for the management of complex stone disease in patients with congenital anomalies. Ultimately, this case illustrates the importance of tailoring surgical approaches to anatomical features rather than relying on standard techniques.
